# Perspective: Beyond the Mediterranean Diet—Exploring Latin American, Asian, and African Heritage Diets as Cultural Models of Healthy Eating^☆^

**DOI:** 10.1016/j.advnut.2024.100221

**Published:** 2024-04-09

**Authors:** Kelly E LeBlanc, Sara Baer-Sinnott, Kristie J Lancaster, Hannia Campos, Ka Hei Karen Lau, Katherine L Tucker, Lawrence H Kushi, Walter C Willett

**Affiliations:** 1 Oldways, Boston, MA, United States; 2 Department of Nutrition and Food Studies, New York University, New York, NY, United States; 3 Department of Nutrition, Harvard University T.H. Chan School of Public Health, Boston, MA, United States; 4 Joslin Diabetes Center, Boston, MA, United States; 5 Department of Biomedical & Nutritional Sciences and Center for Population Health, University of Massachusetts Lowell, Lowell, MA, United States; 6 Division of Research, Kaiser Permanente Northern California, Oakland, CA, United States

**Keywords:** African diet, Latin American diet, Asian diet, traditional foods, nutrition transition

## Abstract

The Mediterranean diet is a well-studied cultural model of healthy eating, yet research on healthy models from other cultures and cuisines has been limited. This perspective article summarizes the components of traditional Latin American, Asian, and African heritage diets, their association with diet quality and markers of health, and implications for nutrition programs and policy. Though these diets differ in specific foods and flavors, we present a common thread that emphasizes healthful plant foods and that is consistent with high dietary quality and low rates of major causes of disability and deaths. In this perspective, we propose that nutrition interventions that incorporate these cultural models of healthy eating show promise, though further research is needed to determine health outcomes and best practices for implementation.

**Statement of Significance**

Despite several calls for culturally appropriate nutrition guidance over the last decade, there has been little research on cultural diets outside of the Mediterranean diet, limiting the evidence base and its utility for informing dietary guidance. The present article comprehensively updates the field by first providing a review of the key characteristics of traditional Latin American, Asian, and African heritage diets, followed by perspectives on best practices for implementation in programs and policy given current evidence.

## Introduction

The Mediterranean diet, with decades of epidemiological and clinical research that support its healthfulness, is a well-studied traditional diet [[1], [2], [3]] that has been recognized by the United Nations Educational, Scientific, and Cultural Organization as intangible cultural heritage of humanity [4], and the Dietary Guidelines for Americans [5] and the WHO [6] as a healthy eating pattern. Yet, research on non-European traditional diets and health outcomes in other cultures with different cuisines has been limited.

To date, there is no universally accepted definition of traditional diets. For the purpose of this review, a traditional or heritage diet is defined as the eating pattern of a particular culture that has been consumed over generations, is aligned with the cultural and/or religious preferences and environmental availability of the region, and prioritizes home-cooked meals featuring biodiverse foods that were commonplace before the advent of highly processed foods and industrial monoculture agriculture.

As traditional diets are displaced by highly processed foods high in refined starches and sugar and sugar-sweetened beverages, noncommunicable diet-related diseases have increased [7]. This phenomenon, known as the nutrition transition, is not inevitable, but it does pose public health challenges [8]. In the United States, dietary acculturation has often been linked with decreased diet quality [[9], [10], [11]]. By calling attention to the diversity of traditional diets that might resonate with individuals in the United States, this perspective aims to inform the United States nutrition community on the value of cultural diets for dietary guidance and areas where further research is warranted.

United States adults of many racial and ethnic backgrounds are disproportionately affected by several diet-related chronic diseases [12]. Not all individuals within a racial or ethnic subgroup share the same national origins, food traditions, or health outcomes, making generalizations across populations difficult. However, the prevalence of health disparities across demographic subgroups warrants attention. As a group, Hispanic Americans have higher prevalence of obesity, type 2 diabetes, and uncontrolled hypertension, but longer life expectancy, compared with non-Hispanic Whites [13]. African Americans have shorter life expectancy than non-Hispanic Whites and are more likely to have obesity, hypertension, diabetes, and risk of stroke [14,15]. Although Asian Americans have less obesity compared with other racial and ethnic groups, the prevalence of obesity is rising faster [16]. Further, Asian Americans are at greater risk of developing type 2 diabetes at a lower BMI (in kg/m^2^) compared with other groups [17].

The root causes of these health inequities include, but are not limited to, structural and systemic racism and social determinants of health, such as inequitable access to quality education, economic stability, quality healthcare, affordable and nutritious foods, and clean air and safe spaces for physical activity [12]. Culture, including people’s customs, traditions, foods, and lifestyle, is also recognized as a social or nonmedical determinant of health that can influence health equity [18]. Training programs to help credentialed nutrition and dietetics practitioners develop cultural competence when working with diverse patient populations exist, though training is inconsistent and not standardized [19].

Although the prevalence and incidence of some diseases, such as type 2 diabetes, differ across populations, available evidence suggests that relationships between diet and disease are similar among different populations, although sometimes to different degrees [20]. For example, among women in the Nurses’ Health Study cohorts, a dietary pattern associated with lower risk of type 2 diabetes in non-Hispanic White women was similarly associated with lower risk of type 2 diabetes among women of African, Asian, and Hispanic ancestry [20].

That said, the foods consumed in different populations can vary greatly due to variation in climate and culture, which makes the study of diet and health important for each of those specific populations. For example, long-term consequences of high levels of beef, dairy, and sugar-sweetened beverage intake may be best studied in Western populations that have had decades-long access to these foods, but studying their health impact in other populations is also warranted. Several dozen large prospective cohort studies in Europe and the United States have provided data on diet and health outcomes for decades, but no such studies exist in Africa or South America [21,22]. Large cohorts in Mexico [23], Japan [24], China [25], and Singapore [26] are now providing data that expand the ranges and types of foods beyond those that are typical of Western populations.

In addition, evidence of the health effects of foods traditionally consumed in understudied settings can support guidelines and recommendations that are culturally appropriate. These are strong reasons to expand research on diet and health beyond its early base in Europe and North America. The aim of this perspective is to summarize the components of traditional Latin American, Asian, and African heritage diets, given their relevance to individuals of diverse racial and ethnic backgrounds in the United States, as well as evidence of their health benefits and implications for nutrition programs and policy in the United States.

## Methods

The search strategy for this narrative perspective was employed to capture a bird’s eye view of the current status of knowledge of these traditional diets and was not designed to systematically grade or quantify the health effects of such diets. A literature search was conducted using PubMed with a focus on English language studies of traditional diets in the 3 regions of interest (Latin America, Asia, and Africa) up to 1 March, 2022. The search terms “traditional Latin American diet,” “traditional African diet,” and “traditional Asian diet” were used in variable order and combinations. In the initial title and abstract review, certain geographic subregions showed up repeatedly in results. Additional narrower search terms, such as “Mexican diet,” “Costa Rican diet,” “Okinawan diet,” and “Japanese diet,” were then employed to capture these additional studies, adding to the overall body of research on these regions rather than limiting the search to these subregions alone.

The literature search was conducted with PubMed because of the focus on health implications of traditional diets. However, to expand the search beyond biomedical and life science articles found in PubMed, and to incorporate research from other social science disciplines, the subject expertise of the authors was also used to identify additional relevant publications. Following removal of duplicates, the titles and abstracts were screened for relevance. Studies that identified key foods or food patterns of traditional diets in the regions of interest and/or the relationship between traditional diets and nutrition and health outcomes, regardless of study design, were included.

To help put traditional diets into a broader context and understand their implications for program and policy, this perspective article refers to some studies capturing the evolution of traditional diets in North America. However, articles on the current-day (postnutrition transition) food patterns of the regions of interest (rather than traditional food patterns) were not the focus of this review and are not included in TABLE 1, TABLE 2 unless the paper indicated some consistency with traditional foods or food patterns.

**TABLE 1:** Key components of traditional dietary patterns throughout Asia, Latin America, and Africa

First author, year	Region of focus	Methods	Key components/characteristics of traditional diet
Kiple, 2007 [27]	Latin America	Archival research	Corn, legumes, fruits, chili peppers, vegetables (potatoes, carrots, zucchini)
Buettner, 2008 [28]	Latin America, Asia	Diet interviews with elders	Legumes (fava, black beans, soy foods, lentils), fruits, vegetables, whole grains, moderate alcohol (wine), limited meat
Momi-Chacón, 2017 [29]	Latin America	Diet interviews with elders	Daily intake of fruits and vegetables, black beans, corn tortillas, white rice, “gallo pinto,” dairy products, and “fresco”; Lower intake of red meat, sweets, salty snacks
Santiago-Torres, 2015 [30]	Latin America	Food frequency questionnaire modified with Hispanic foods	Corn tortillas, beans, soups, mixed dishes, fruits, vegetables, full-fat milk, cheese; low in refined grains and added sugars
Navarro, 2010 [31]	Latin America	Narrative review	Legumes, starches (corn, yucca), fruits (pineapple, passion fruit, avocado), chili peppers, vegetables (zucchini, sweet potato), small amounts of meat
Defagó, 2021 [32]	Latin America	Food frequency questionnaire	Fruits, vegetables, legumes, whole grains, fish, seafood and nuts
Orona-Tamayo, 2019 [33]	Latin America	Narrative review	Legumes, corn, amaranth, chia seeds, quinoa
Oldways, n.d. [34]	Latin America	Subject-matter expert opinion recommendations	Daily intake of fruits, vegetables, whole grains, beans, nuts, legumes, seeds, herbs, and spices; moderate intake of fish, seafood, poultry, eggs, cheese, and yogurt; minimal intake of meats and sweets
Whitton, 2018 [26]	Asia	Food frequency questionnaire	Whole grains, dairy, fruit, vegetables, unsaturated cooking oils; low intakes of fast food, sugar-sweetened beverages, poultry, processed meats
Lukito, 2001 [35]	Asia	Narrative review	Frequent intake of soy foods (tofu, tempeh, soy drinks and soy desserts), lentils, and nuts
Dixit, 2011 [36]	Asia	Narrative review	Rice (white and brown); ancient whole grains like barley, pearl millet, finger millet, sorghum,
Rodzi, 2021 [37]	Asia	Narrative review	Fermented foods (such as nham, tempoyak, sayur asin, prahok, bagoong, padack, laphet, and dua gia)
Willcox, 2014 [38]	Asia	Narrative review	Root vegetables, green and yellow vegetables, soy foods, medicinal plants; moderate amounts of marine foods, fruit, spices, tea, alcohol
Kromhout, 2018 [39]	Asia	7-d diet recall diary, subsample of 7-d weighed food sampling	Vegetables, starch; limited sweets, animal foods, saturated fat
Tsugane, 2014 [24]	Asia	Food frequency questionnaire	Soy foods, seafood, green tea, salted foods, low intake of red meat and saturated fat
Tomata, 2019 [40]	Asia	Food frequency questionnaire	Rice, miso soup, seaweed, pickles, green and yellow vegetables, fish, green tea; limited beef and pork
Tsugane, 2021 [41]	Asia	Narrative review	Low intake of red meat, high intakes of fish, soy foods, unsweetened beverages like tea
Wang, 2019 [42]	Africa, Asia, Latin America	24-h diet recall	Great variability in diet depending on country/region
Chen, 2006 [25]	Asia	3-d weighed household survey	Vegetables and plant foods (soy foods, legumes, grains, fruits)
Chen, 1990 [43]	Asia	3-d diet record	Vegetables and plant foods (soy foods, legumes, grains, fruits)
Micha, 2015 [44]	Asia, Africa	Diet records, food frequency questionnaires, and household availability surveys	Great variability in diet depending on country/region
Oldways, n.d. [45]	Asia	Subject-matter expert opinion recommendations	Daily intake of fruits, vegetables, whole grains, beans, nuts, legumes (including soy foods), seeds, herbs, and spices; moderate intake of fish, seafood, poultry, eggs, cheese, and yogurt; minimal intake of meats and sweets
Reicks, 2022 [46]	Africa	Food behavior survey	Daily intake of fruits, vegetables (including greens), whole grains, beans, nuts, legumes, seeds, herbs, and spices
da Silva, 2017 [47]	Africa	Archival research	West and Central African crops, such as collard greens, black-eyed peas, okra, watermelon
Miller, 2013 [48]	Africa	Archival research	Cornbread and cornmeal, leafy greens, yams and tubers, seafood, poultry and organ meats
Carney, 2001 [49]	Africa	Archival research	Rice
Harris, 2010 [50]	Africa	Archival research and interviews with elders	Leafy greens, cassava, grains (millet, corn, couscous), fruits (like mango), mint tea, water, beans and peas, vegetables (including squash), fish, spicy condiments, meats
Harris, 2012 [51]	Africa	Archival research and interviews with elders	Grain foods (corn, cornbread, millet, fonio, porridges), leafy greens, tubers (like yams), okra, watermelon, legumes (peanuts, black-eyed peas), meat, fish
Rousseau, 2018 [52]	Africa	Archival research	Fruits and vegetables (such as ackee, cho cho, peppers, eggplant, okra, hearts of palm, artichoke, pumpkin, banana, plantain, mango, papaya, melon, guava), greens (such as callaloo, pak choi, lettuce, arugula, cabbage, broccoli, cauliflower), peas and beans, grains, coconut)
Spivey, 1999 [53]	Africa	Archival research	Rice, legumes (such as peanuts, black-eyed peas, and other beans), vegetables (such as okra and eggplant), nuts, fruits (like pineapple and oranges), hot peppers and spices, cassava, fish and game meat
Sodjinou, 2009 [54]	Africa	3 nonconsecutive 24-h diet recalls	High in fiber and vegetables, low in fruit, fat, sugar, and cholesterol
National Research Council, 1996 [55]	Africa	Survey of scientists and organizations across Africa	Grains (including sorghum, fonio, millets, teff, and rice)
Dunne, 2022 [56]	Africa	Archaeobotanical and organic residue analysis	Leafy greens, pulses, grains, root vegetables
Oldways, 2018 [57]	Asia	Subject-matter expert opinion recommendations	Daily intake of fruits, vegetables, whole grains, beans, nuts, legumes, seeds, herbs, and spices; moderate intake of fish, seafood, poultry, eggs, cheese, and yogurt; minimal intake of meats and sweets

**TABLE 2:** Traditional dietary patterns throughout Asia, Latin America, and Africa and their relationship with nutrition and health outcomes

First author, year	Region of focus	Participants (individuals, unless otherwise noted)	Dietary assessment method	Key components/characteristics of traditional diet	Outcomes associated with traditional diet/dietary components
Medina-Inojosa, 2014 [58]	Latin America	*n* = 13 studies	Not reported	Legumes, fruits	Lower risk of mortality despite higher risk of cardiovascular disease risk factors
Buettner, 2008 [28]	Latin America, Asia	Not reported	Diet interviews with elders	Legumes (fava, black beans, soy foods, lentils), fruits, vegetables, whole grains, moderate alcohol (wine), limited meat	Lower risk of mortality
Rosero-Bixby, 2013 [59]	Latin America	*n* = 16,300	Food frequency questionnaire	High fiber foods (vegetables, fruits, legumes), rice, animal proteins, limited milk	Greater life expectancy, lower risk of cardiovascular disease risk factors, longer telomeres
Momi-Chacón, 2017 [29]	Latin America	*n* = 34	Food frequency questionnaire	Mostly plant-based foods including grains and legumes, smaller amounts of meat, dairy, and fermented beverages	High diet quality
Kabagambe, 2005 [60]	Latin America	*n* = 4238	Food frequency questionnaire	Beans	Lower risk of nonfatal acute myocardial infarction
Mattei, 2011 [61]	Latin America	*n* = 1879	Food frequency questionnaire	Beans, rice	Lower risk of cardiometabolic risk factors
Curi-Quinto, 2022 [62]	Latin America	*n* = 2438	7-d semiquantitative food frequency questionnaire	Higher in whole grains and beans; lower in sodium, added sugars, saturated fats	High diet quality
Santiago-Torres, 2015 [30]	Latin America	*n* = 493	Food frequency questionnaire	Corn tortillas, beans, soups, mixed dishes, fruits, vegetables, full-fat milk, cheese; low in refined grains and added sugars	High diet quality, lower serum C-reactive protein and insulin concentrations
McMurry, 1991 [63]	Latin America	*n* = 13	Crossover dietary intervention	Pinto beans, corn, fruit, vegetables, chili peppers, coffee, small amounts of sugar and egg whites	Lower coronary risk factors (plasma lipid, lipoprotein, weight)
Navarro, 2010 [31]	Latin America	*n* = 854	Food frequency questionnaire	Legumes, starches (corn, yucca), fruits (pineapple, passion fruit, avocado), chili peppers, vegetables (zucchini, sweet potato), small amounts of meat	Lower blood pressure, cholesterol, and diabetes risk
Sadeghi, 2019 [64]	Latin America	*n* = 700	Nutrition education intervention	Fruits and vegetables	Reduced rate of BMI growth
Hu, 2016 [65]	Latin America	*n* = 186	Nutrition education intervention	Whole grains, non-starchy vegetables; limited sodium, total and saturated fat, portion sizes, refined carbohydrates, starchy vegetables	Improved diabetes knowledge, diabetes self-efficacy, and blood sugar control (as measured by HbA1C)
Tsugane, 2014 [24]	Asia	*n* = 130,000	Food frequency questionnaire	Soy foods, seafood, green tea, salted foods, low intake of red meat and saturated fat	Lower risk of certain cancers, cardiovascular disease, and diabetes for some dietary components, higher risk of certain cancers, cardiovascular disease, and diabetes for other dietary components
Okada, 2018 [66]	Asia	*n* = 58,767	Food frequency questionnaire	Legumes, seafood, vegetables, pickles, fungi, seaweeds, fruits	Lower risk of all-cause and cardiovascular disease mortality
Ozawa, 2013 [67]	Asia	*n* = 1006	Food frequency questionnaire	Soy foods, vegetables, algae, dairy, limited rice	Lower risk of all-cause dementia, Alzheimer’s dementia, and vascular dementia
Hsu, 2014 [68]	Asia	*n* = 50	Randomized controlled trial	High fiber, high carbohydrate foods; limited fat	Lower insulin resistance
Galbete, 2017 [69]	Africa	*n* = 3905	Food Propensity Questionnaire and subsample of 24-h diet recalls	Root vegetables, tubers, plantains, fermented corn products	Lower BMI
Nkondjock, 2010 [70]	Africa	*n* = 571	Food frequency questionnaire	Fruits, vegetables, tubers, legumes	Lower risk of hypertension
Agurs-Collins, 2009 [71]	Africa	*n* = 50,778	Food frequency questionnaire	Whole grains, vegetables, fruit, fish	Lower risk of breast cancer
O’Keefe, 2015 [72]	Africa	*n* = 40	controlled dietary intervention	Corn, seafood, leafy greens, fruit (bananas, mango, pineapple, guava), lentils, beans	Reduced colonic inflammation, increased diversity of gut microbes, lower production of bile acids
Reicks, 2022 [46]	Africa	*n* = 586	Food behavior survey	Daily intake of fruits, vegetables, whole grains, beans, nuts, legumes, seeds, herbs, and spices	Decreased weight, waist circumference, and systolic blood pressure; diet quality (increased intake of fruit, greens, and total vegetables)

Abbreviation: HbA1C, glycated hemoglobin.

Table 1 describes the 34 studies [[24], [25], [26], [27], [28], [29], [30], [31], [32], [33], [34], [35], [36], [37], [38], [39], [40], [41], [42], [43], [44], [45], [46], [47], [48], [49], [50], [51], [52], [53], [54], [55], [56], [57]] describing the key characteristics of traditional diets across the 3 regions of interest. Table 2 describes the 21 studies [24,[28], [29], [30], [31],^46^,[58], [59], [60], [61], [62], [63], [64], [65], [66], [67], [68], [69], [70], [71], [72]] detailing the relationship between traditional diets and nutrition and health outcomes. Given the wide-ranging and interdisciplinary scope of this perspective, a narrative synthesis of the results was performed with no quantitative analysis.

## Discussion

### Exploring traditional diets

True to many agrarian societies throughout history, traditional Latin American, Asian and African heritage diets tend to follow a “core-fringe-legume” pattern [73], consisting of unrefined carbohydrate foods, like whole grains or tubers, as the base (core) of the meal, along with vegetables and small amounts of meats, sauces, or fish (fringe) and legumes, which add flavor and variety.

Historically, not all elements of traditional diets were universally health-promoting. For example, in the 1960s, the Seven Countries Study found that incidence of coronary heart disease was ∼10 times higher in Finland compared with that in 2 Japanese villages or in Crete [74]. In China, the prevalence of coronary heart disease differed many-fold between northern China, where harsh climates limited production of fruits and vegetables, and southern China, where these foods were abundant [75]. Many traditional diets were also influenced by the need to preserve food across cold or dry seasons and, for this purpose, some used large amounts of salt, which substantially increases risk of cardiovascular disease [76].

Cultural food preferences also strongly influence diets. In many regions, highly refined white grains have been associated with prestige but have deleterious effects on nutrition and health because much of the nutritional value has been stripped away [36,77]. For these reasons, many traditional diets include elements that are health-promoting and should be emphasized, and other elements that may be less healthy and, therefore, deemphasized.

Sufficient evidence has accrued to identify dietary components that are likely to enhance or to compromise health and wellbeing [78,79]. This knowledge has been increasingly used to create diet quality scores, such as the Mediterranean diet score [80], Healthy Eating Index [81], Alternative Healthy Eating Index (AHEI) [82], and Dietary Approaches to Stop Hypertension score [83]. These scores predict mortality and a wide variety of other health outcomes across different cultures [84,85], although studies among populations of Asia, Africa, and Latin America remain limited. Notably, when rated by a modified version of the AHEI that excludes alcohol, dietary quality in some counties in Asia, Africa, and Latin America had scores that were similar to those of the Mediterranean region, although the highest scores were ∼65 out of 100, indicating potential for improvement in all regions [42] in the context of dietary guidance developed for the United States population.

The following subsections describe elements of traditional Latin American, Asian, and African heritage diets (consumed both within their places of origin and within the United States) and evidence on their relation to health outcomes. Although it is not possible to adequately portray the multitudes of diet variation within continents, these subsections highlight the commonalities within each region, whereby an overall pattern emerges.

### Traditional food patterns across Latin America

Individuals of Latin American descent are heterogeneous in their dietary habits and food traditions, given the diversity of cultures, geographies, and life experiences of these populations. Rather than portray a singular diet to represent all individuals in this population, this perspective explores Latin American heritage diets more broadly, emphasizing the wider eating pattern. The Latin American eating pattern is a variable blend of the broad traditional diets of pre- and post-Columbian cultures, including those of indigenous peoples (Aztecs, Incas, Mayans, and other Native Americans) and influences from Spanish, Portuguese, and continental Africans [27].

Although substantial variation in diets exists within this broad geographic area, traditional diets in Latin America tend to be rooted in whole grains (primarily maize) and beans [28], along with ample fruits and vegetables (like peppers, tomatoes, avocado, potatoes, pineapple, passion fruit, carrots, and zucchini) [[29], [30], [31]] and sometimes seafood [32]. Many ingredients popularized as “superfoods” today, such as quinoa, amaranth, chia seeds, and acai berries, are native to Central or South America [33].

**FIGURE 1:**
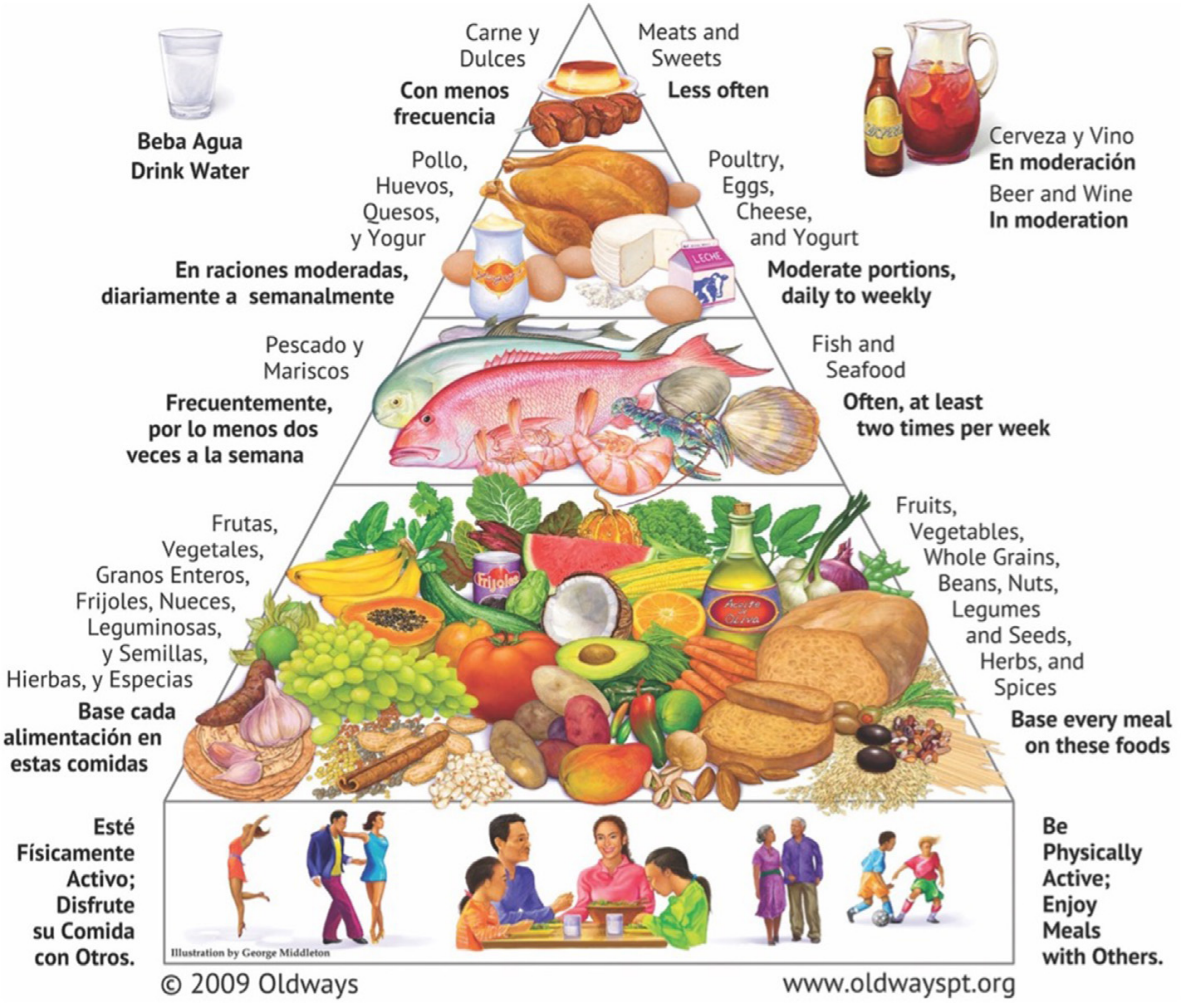
Latin American Diet Pyramid. Figure reproduced with permission from Oldways [34].

The elements of a healthy Latin American heritage diet are illustrated in graphic form by the Oldways Latin American Heritage Pyramid, which was first introduced at the 1996 Latin American Diet Conference and then updated with new graphics in 2009, following input from the 2005 Latin American Diet Summit [34] (Figure 1). Unlike a plate graphic, which illustrates a single meal, this Pyramid graphic was selected to illustrate the wide heterogeneity of foods that exist within this broader dietary pattern.

### Health outcomes of traditional Latin American diets

As a group, Hispanic Americans live longer and have lower rates of heart disease than non-Hispanic Whites, despite higher prevalence of risk factors for heart disease and mortality, possibly due, in part, to their traditional diet and/or high level of social and familial support [58].

An investigation of the lifespan and lifestyle habits of the population of Costa Rica’s Nicoya Peninsula, a hot spot of high longevity [28], noted a prevalence of foods with low glycemic indices and high levels of dietary fiber. This suggests adherence to a traditional diet, with relatively low intake of processed foods and high intakes of beans, fruits, and vegetables [59]; 82% of the long-lived residents of Nicoya reported eating black beans 1–3 times per day; 74% reported eating fruits, vegetables, and tortillas 1–3 times per day; and no one reported daily intake of red meat [29]. Higher intake of black beans was associated with higher HDL cholesterol; lower systolic and diastolic blood pressure, fasting glucose, and triglyceride concentrations; and lower risk of metabolic syndrome and coronary heart disease in the Costa Rica Heart Study, which used dietary data from 2004 [60,61]. The value of traditional diets was also demonstrated in a recent analysis from Mexico; diet quality was higher and food cost was lower in rural indigenous areas than in urban areas [62].

Lower inflammation may mediate health benefits from traditional diets. Among Mexican women, a traditional Mexican Diet Score (higher intakes of corn tortillas, beans, soup, fruits, vegetables, whole grains, and Mexican cheese and lower intakes of added sugars, reﬁned grains, and added fats) was associated inversely with blood concentrations of the inflammation marker, high-sensitivity C-reactive protein, and insulin [30]. In an early trial among an indigenous population of Mexico following a traditional dietary pattern, blood lipid patterns changed adversely when they were fed a Western diet [63]. Similarly, lower blood cholesterol, blood pressure, and type 2 diabetes risk were seen among traditional Latin American populations consuming a largely plant-based diet including beans, corn, squash, sweet potato, and fruits throughout Brazil, Mexico, and the Peruvian Highlands [31].

The principles of Latin American heritage diets, mainly tailored to the Mexican American subgroup, have been incorporated into intervention programs in the United States with modest success. For example, the Niños Sanos, Familia Sana nutrition intervention in California’s Central Valley resulted in reduced rate of BMI growth among Mexican-heritage children; more research is needed on how to sustain these improvements for longer periods or how these lessons might be adapted for other United States Hispanic subgroups [64]. Lifestyle and nutrition programs culturally tailored to Latin American heritage diets have also shown promise in improving diabetes knowledge, diabetes self-efficacy, and blood sugar management (as measured by glycated hemoglobin) among United States Hispanics with type 2 diabetes and their families [65].

### The evolution of Latin American food patterns in North America

Great diversity exists across United States Hispanic subgroups, and a longer life expectancy is not uniform. Puerto Ricans, even on the island, tend to have premature mortality [86] and poor-quality diets [87], probably because the island’s nutrition transition occurred much earlier than among other Latin American populations. While some migrants from Latin American communities retain healthful aspects of their traditional dietary patterns, other migrants risk poor nutrition as they shift toward a more Western diet filled with highly processed foods, sugar-sweetened beverages, and foods with added solid fats. For example, in 2012, Mexican Americans had the highest AHEI score among ethnic groups in the United States, and the higher dietary quality of Mexican Americans, compared with that of non-Hispanic Whites, was not attributable to differences in socioeconomic status [88]. The Hispanic Community Health Study/Study of Latino Youth found that youth who report remaining integrated in Latino culture (rather than fully assimilated into American culture) may eat more whole grains and consume a lower percentage of energy from solid fats and added sugars [89]. However, ﬁrst generation United States children are likely to abandon their traditional Mexican diet for “American” highly processed foods containing added sugars and solid fats, lowering their diet quality [90].

### Traditional food patterns across Asia

It would be inaccurate to suggest that a single diet could adequately convey the eating patterns of the most populous continent in the world, as specific food traditions vary widely between and within countries, cities, and households. Instead, by zooming out to analyze the overall pattern of Asian heritage diets, this perspective highlights common dietary building blocks that appear throughout the region. Despite diﬀerences among the cultures in East, Southeast, and South Asia, many traditional Asian diets share a common eating pattern of vegetables [26]; vegetarian protein sources like tofu, legumes, or nuts [35]; whole grains such as millet and barley [26,36]; and fermented foods [37].

Okinawa, Japan has a large proportion of people who live healthfully into their 90s and 100s [28]. The traditional diet in Okinawa emphasizes root vegetables (principally sweet potatoes), green and yellow vegetables, soybean-based foods, and medicinal plants, with marine foods, lean meats, fruit, medicinal garnishes and spices, tea, and alcohol consumed in moderation [38].

Some authors have argued that the overall eating patterns of traditional Japanese diets are similar to Mediterranean diet patterns—high in vegetables and fish and low in sweets and meats [38,39]. However, others have noted that a weakness of the Japanese diet is its high salt intake from soy sauce, miso, pickled vegetables, and salted seafood [24]. The Japanese Diet Index, a measure of how well participants’ diets align with a traditional Japanese diet, is correlated favorably with all 12 nutrients studied except for sodium [40]. The Japanese diet of recent years is of particular interest because life expectancy in Japan has improved steadily and is now the longest globally, ∼5–6 y longer than that in the United States [41]. A partial Westernization of the Japanese diet, with a greater variety of foods displacing some of the large amounts of white rice consumed in earlier decades, may have contributed in part to this exceptional longevity [41]. The result of this shift is a dietary composition similar to a traditional Mediterranean diet but with foods and flavors of Japan [38,39]. The AHEI dietary quality score of Japan is among the highest globally and is far higher than that of the United States [42].

Many cuisines across Asia boast a similar history of healthy, mostly plant-based diets, although great variability exists in the specific foods from one region to the next [38]. In ecological analyses within China, primarily plant-based diets were associated with numerous positive health outcomes [25,43]. A cross-sectional, multiethnic cohort study in Singapore identified a shared healthy eating pattern among a subset of Chinese, Malay, and Indian cultures, which is based on fruits, vegetables, dairy, wholegrain breads, breakfast cereals, unsaturated cooking oils and is low in fast food, sweetened beverages, meat, and ﬂavored rice (rice that is not served plain, e.g., fried rice) [26].

Today, a diversity of healthful food traditions continue to exist in nations across Asia. Despite high sodium intake, diets of several Eastern Asian countries/territories in addition to Japan, including Taiwan, Vietnam, Cambodia, and the Republic of Korea (South Korea), had relatively high AHEI scores, suggesting some success preserving the healthful components of their traditional diets [42].

These findings were also reflected in the Global Dietary Database, an analysis of modern-day (2010) food patterns [44]. Specifically, the Global Dietary Database found that Southeast Asian nations (Cambodia, Malaysia, Myanmar, Laos, Vietnam) had particularly high intakes of nuts and seeds, and high-income Asia Pacific and East Asian countries had high intakes of vegetables. While white rice is often seen as the staple grain of Asia, intake of whole grains (such as whole grain breads and cereals) was high in several South and Southeast Asian nations, such as Pakistan, Bangladesh, Malaysia, Cambodia, and Myanmar. Intakes of unprocessed red meat and processed meat were low in India, Sri Lanka, Bangladesh, Pakistan, and Singapore. The island and coastal nations of Japan and South Korea had some of the highest seafood intakes. In the period from 1990 to 2010, South Asia was the only region that experienced a small but statistically significant increase (+3.8 g/d) in fruit consumption. Increases in nut and seed consumption were seen in both Southeast Asia (+11 g/d) and South Asia (+3.2 g/d). Central, East, and Southeast Asia also experienced slight increases in seafood consumption (2.8–4.2 g/d). However, significant decreases in whole grain consumption were also noted in South Asia (−12.1 g/d) and East Asia (−6.8 g/d), and significant increases in unprocessed red meat consumption were noted in East Asia (+8.3 g/d) [44].

**FIGURE 2:**
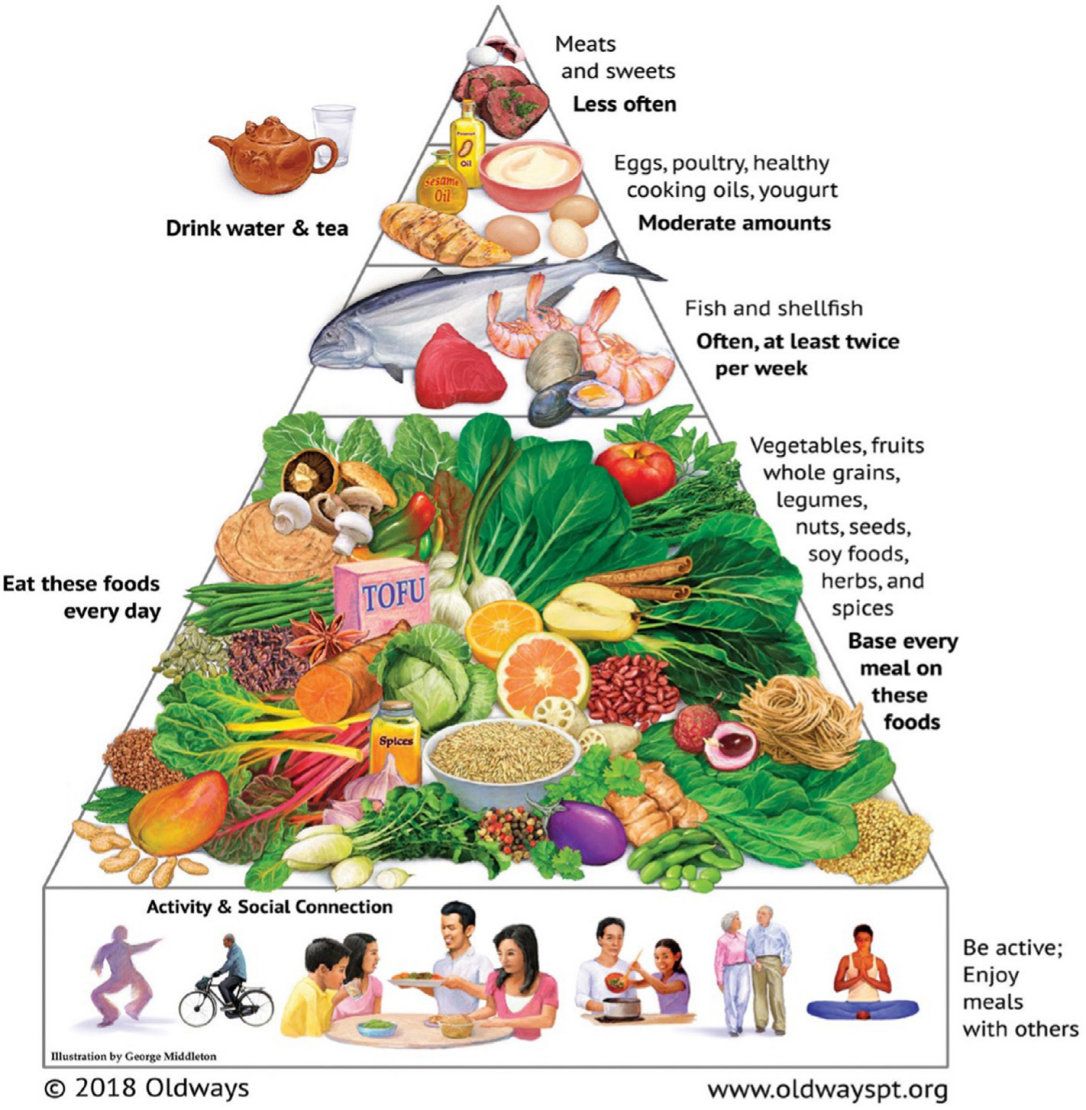
Asian Diet Pyramid. Figure reproduced with permission from Oldways [45].

The Oldways Asian Diet Pyramid, which was first introduced at the 1995 International Conference on the Diets of Asia in collaboration with the Harvard School of Public Health and Cornell University and then updated in 2018 following input from the Oldways Asian Diet Scientific Advisory Committee [45], was selected to further illustrate the substantial variation of foods and flavors within the broader Asian heritage diet (Figure 2).

### Health outcomes related to traditional Asian diets

Diets featuring traditional Asian foods, especially those higher in fiber, show promise for preventing chronic disease. The Japan Public Health Center Study found associations between numerous aspects of a typical Japanese diet (e.g., high intakes of soy foods and isoflavones, fish and n–3 fatty acids, and green tea, and low intakes of red meat and saturated fat) and lower risks of certain cancers, cardiovascular disease, and type 2 diabetes [24]. Nonetheless, the Japan Public Health Center Study also noted that excessive sodium intake in Asian diets poses risk for cardiovascular and other noncommunicable diseases. In a cohort study of 58,767 adults in Japan, participants with the highest Japanese Food Score had significantly lower risks of all-cause and cardiovascular mortality but not cancer mortality [66]. A diet “roughly correspondent to a customary Japanese diet” high in soy, green vegetables, and algae was linked with significantly lower risk of dementia in an Asian population [67]. Another randomized controlled pilot study showed that a traditional Asian diet (70% carbohydrate, 15% protein, 15% fat) centered on vegetables, whole grains, and primarily plant-based protein may help in lowering insulin resistance, a risk factor for type 2 diabetes [68].

### The evolution of Asian dietary traditions in North America

While globalization has made it easier to recreate traditional dishes anywhere, sodium continues to be a nutrient of concern among Asian-descendant populations. Asian Americans have higher sodium intake than Americans of other racial and ethnic backgrounds, which tends to be from cooking rather than from packaged and restaurant foods [91]. In addition, white rice is often consumed in large amounts, displacing ancient traditional whole grains like brown rice, barley, and millet [36], which can contribute to risk of type 2 diabetes [92].

The nutrition transition toward refined grains and sugar-sweetened beverages, juices, and snacks may also pose health risks. A cross-sectional study found that Canadians of South Asian descent had the highest consumption of sugar-containing soft drinks, juices, and snacks, compared with Canadians of European, Indigenous, or Chinese descent, and that higher intake of these foods was associated with lower HDL cholesterol [93].

### Traditional food patterns across the African diaspora

Specific cultural dishes vary widely from one part of the African diaspora to the next. Rather than describe one single unifying diet to represent all African-descendant individuals, the term African heritage cuisine is used more broadly in this perspective to describe the traditional foods brought to the New World by Africans, along with those they adopted. Great culinary and geographic diversity exists, as African heritage foods are shaped by 4 major regions: continental Africa, the American South, the Caribbean, and South America [46].

During the Trans-Atlantic Slave Trade, people from Western and Central Africa were forcibly abducted from their lands and taken to the Americas, creating the new ethnic group of African Americans [47]. Subsequently, in the United States context, the Great Migration (1915–1970), was characterized by nearly 6 million Black Americans moving from the South to the North and the western United States. Thus, African heritage foods were transported across the United States and continued to shape American cuisine [48].

Many African American cultural foods, such as collard greens, black-eyed peas, and okra, have their roots in West and Central Africa [47,48]. Enslaved people, particularly women, provided the knowledge and skills for rice cultivation, which remains an enduring staple crop throughout the Americas today [49]. In addition to cultivating and popularizing foods from Africa, early African American food staples were shaped by local vegetables growing in enslaved people’s private gardens, foraged from the wild, or served in Southern plantation houses [50,51].

Afro-Caribbean cuisine draws its origins from the arrival of African captives to the sugarcane plantations of the West Indies and the Caribbean. There, African staple foods like okra, yams, and greens were combined with tropical accents, peppery sauces, and various seafoods [51,52]. Afro-South American cuisine, which developed when Africans were brought to South America (especially Brazil), has influences from Spain, Portugal, and indigenous peoples. This is evident in the prominence of rice and bean dishes, fish (such as pataka) and vegetables stews, and root vegetables (like cassava) [53].

Research suggests that these African heritage diets are of high dietary quality and may be linked with health benefits. In Benin (West Africa), a healthful traditional dietary pattern was identified, with high intake of fiber and grains (often maize with vegetables) and fruit and lower amounts of saturated fat, sugar, and cholesterol than the transitional dietary pattern (featuring more dairy, meats, sweets, bread, and eggs) [54]. Teff, millet, and sorghum are also important cereal grains in Africa and are more nutritionally dense than refined cereals [55]. Leafy vegetables (greens), cereals, legumes, and yams are also key elements of traditional West African diets [56].

Despite the ongoing nutrition transition, many healthful habits persist. In an analysis of modern dietary patterns, fruit intake was high in Caribbean nations, and intakes of whole grains, vegetables and legumes were high in Sub-Saharan Africa [44]. This study also found that seed and nut intake varied throughout Africa, with higher intakes in western Sub-Saharan Africa and lower intakes in southern and eastern Sub-Saharan Africa. Between 1990 and 2010, Central Sub-Saharan Africa and North Africa/Middle East were among the many regions to experience significant decreases in whole grain consumption, at −65.9 g/d and −8.1 g/d, respectively. When rated by the AHEI, many North and West African diets were of similar dietary quality to those in Mediterranean and healthy East Asian regions [42].

**FIGURE 3:**
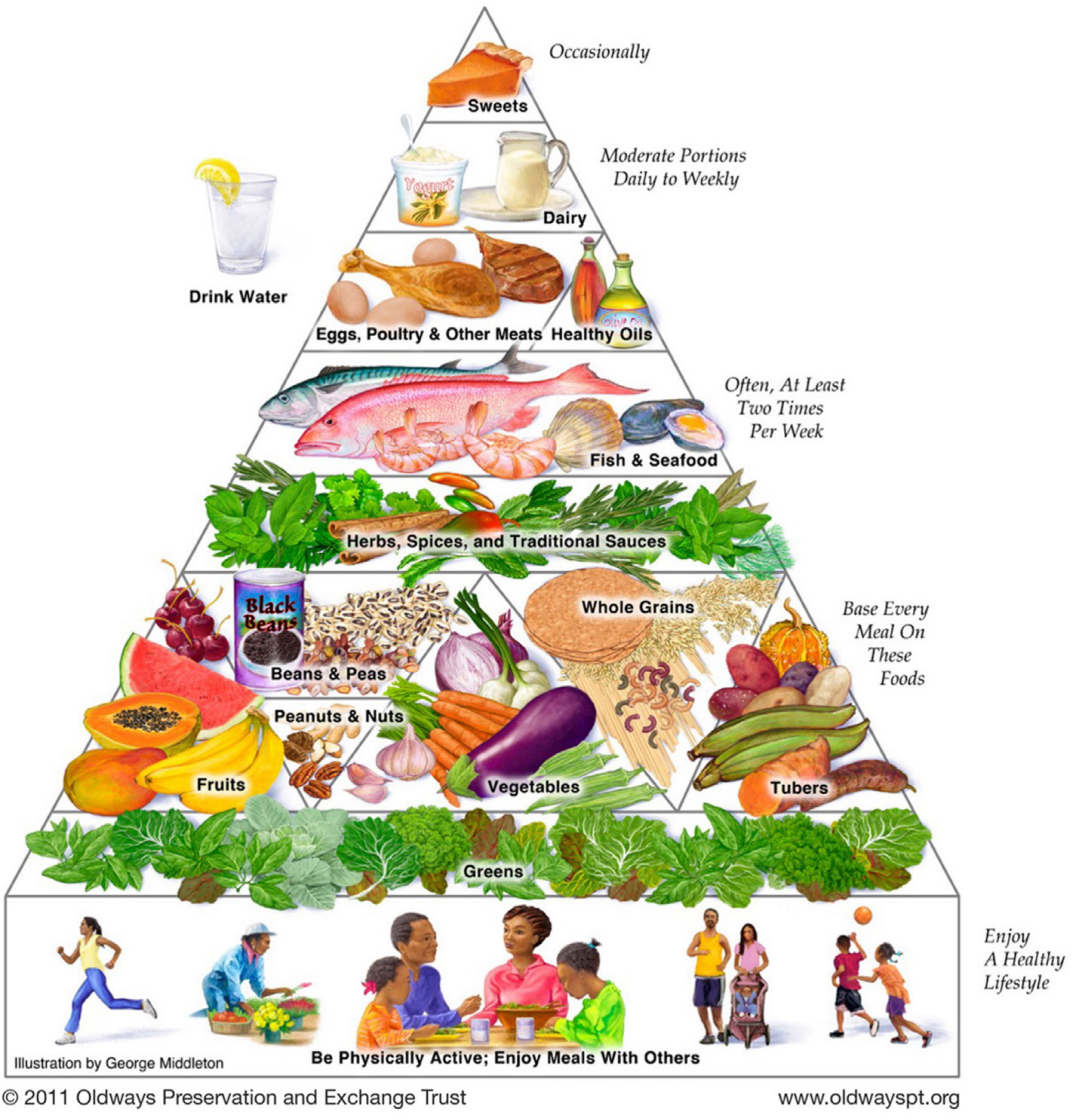
African Heritage Diet Pyramid. Figure reproduced with permission from Oldways [57].

The elements of a healthy African heritage diet are illustrated in graphic form by the Oldways African heritage pyramid, which was created by nutrition nonprofit Oldways in 2011 with the help of nutrition scientists, health experts, and culinary historians [57] (Figure 3). Because one single diet or plate does not fit all African Americans, this Pyramid graphic was selected to represent the substantial diversity of ingredients that exist within the broader African heritage dietary pattern.

### Health outcomes related to traditional African diets

In a study of 4543 Ghanaians living in urban Ghana, rural Ghana, and Europe, those living in rural Ghana tended to eat more roots, tubers, plantains, and fermented corn products, and they also had the lowest BMI [69]. Among military personnel in Cameroon, those eating a diet rich in fruit, vegetables, tubers, and legumes had significantly lower risk of hypertension than those eating few fruits and vegetables [70]. Within an African American population, women who consumed a dietary pattern consistent with an African heritage diet experienced lower risk of breast cancer, especially the more aggressive estrogen receptor-negative form, than those who did not follow this pattern [71].

African heritage diet intervention studies are sparse but promising. O’Keefe et al. [72] assigned 20 middle-aged African Americans to a traditional African heritage diet (averaging 55 g ﬁber daily and 16% energy from fat, with foods like mangos, bean soup, and ﬁsh) and 20 middle-aged rural South Africans to a typical American diet (averaging 12 g ﬁber daily and 52% energy from fat, with foods like pancakes, burgers, fries, and meatloaf). In 2 wk, the African Americans reduced colonic inﬂammation, improved risk markers for cancer, and increased the diversity of their healthy gut bacteria, while the rural Africans eating an American diet showed greater production of bile acid and decreasing diversity of healthy gut bacteria [72].

In a sample of 586 adults across 21 states, participation in a 6-wk cooking and nutrition curriculum, based on healthy plant-based foods from across the African diaspora, was associated with decreased weight, waist circumference, and systolic blood pressure, as well as increased intakes of fruit, greens, and total vegetables [46]. More research is needed to better understand the relationship between African heritage diets and health outcomes.

### The evolution of African-descendant diets in North America

Although African American ancestors introduced many healthy crops into the American diet, the legacy of slavery and systemic racism denied African Americans equitable access to many of these foods and continues to do so to this day [94]. Cultural preferences are not always a predictor of food choice—socioeconomic status and food access play an important role, particularly given the prevalence of inexpensive, unhealthy foods in high-income countries. Among ethnic groups within the United States, African Americans had the lowest AHEI score, but this difference was largely explained by socioeconomic inequity [88]. Black and Hispanic Americans are also disproportionately targeted by advertising for unhealthy food and beverages compared with non-Hispanic White Americans [95].

Immigration to nations with a Western food culture (e.g., with sugar-sweetened beverages and snacks with added salt, solid fat, and sugar) is also related to dietary quality. For example, among Haitian immigrants to Canada, overall diet quality was signiﬁcantly healthier in the Haitians consuming the traditional diets than the Western diets, and duration of residence in Canada was associated with higher intakes of total fat and cholesterol [96].

### Implications for practice and policy

In recent decades, populations around the world have transitioned from traditional diets to diets including inexpensive, rapidly prepared, highly processed foods, and sugar-sweetened beverages that are high in energy and low in nutritional value [97,98]. The public health challenges arising from this shift have been well documented in the literature [[99], [100], [101], [102], [103], [104]].

The overall pattern of traditional Asian, Latin American, and African heritage diets share many characteristics, with an emphasis on beans and whole grains supplemented with ample fruits and vegetables and accented with smaller portions of fish, poultry, and meats. These patterns also share commonalities with the dietary patterns recommended in the 2020–2025 Dietary Guidelines for Americans, which consist of ample vegetables, fruits, legumes, and whole grains and limited sources of saturated fats and sugars [5]. Indeed, these traditional patterns may be thought of as different culturally-celebratory approaches to achieve the objectives of dietary guidelines to promote health. However, these traditional dietary patterns tend to be less animal-centric (i.e., less meat and dairy) than the United States–style dietary pattern in the 2020–2025 Dietary Guidelines for Americans, a distinction with important environmental implications [79].

These traditional dietary patterns, in turn, provide the framework to describe the more specific foods eaten in a particular country or region. Thus, traditional models of healthy eating provide culturally appropriate, personalized paths to improve diets for Americans of diverse backgrounds, even as they incorporate nutritional principles gained from the extensive research on the Mediterranean diet. For example, a meta-analysis of 28 randomized controlled trials in different ethnic minority groups found that culturally appropriate health education improves short- and medium-term glycemic control in people with type 2 diabetes [105]. Strategies to help children and adults retain their healthy traditional food cultures may continue to be an important area of focus for nutrition professionals.

In a study of Mexican-heritage households in California’s Central Valley, fruits and vegetables with cultural significance were among the most highly purchased foods, suggesting value in including such foods in assistance programs [106]. In one study in the United States South, foods described as Southern were perceived to taste better [107], suggesting that making a cultural connection with a healthy food may increase the likelihood of acceptance. Future research should apply these findings to nutrition assistance program pilots to measure how tailored education featuring traditional diets may improve dietary quality in participants of the Supplemental Nutrition Assistance Program, the National School Lunch Program, or other nutrition programs.

### Strengths and limitations

The racial and ethnic makeup of the dietetics profession in the United States is disproportionately non-Hispanic White [108], and there is substantial interest among dietitians to advance professional knowledge of diverse cultural food traditions to reduce health disparities in diverse patient populations [19]. A strength of this study is that it discusses how evidence from traditional dietary patterns comprised of culturally appropriate foods offer beneficial foods and nutrients that are already in alignment with long-standing and current dietary guidelines for Americans. This perspective also calls attention to a much-needed area of diet and health research beyond its early base in Europe and North America by shedding light on the traditional eating patterns of Asian heritage, Latin American heritage, and African heritage.

Given the scope of this perspective article, numerous limitations must be acknowledged. One is the paucity of research on traditional diets in the 3 regions of interest, especially outside of Japan, China, India, Costa Rica, and Mexico. Given the unequal geographic distribution of the studies, the evolving concepts of what self-identified race or ethnicity means throughout the literature, and the variation of how different cultural groups are defined in different studies, the results on the health benefits of a particular heritage diet may not be able to be extrapolated to each and every subgroup within that heritage. Given the heterogeneous sources and study designs examining the nutrition and health outcomes of traditional diets (Table 2), it was not possible to assess the statistical significance of each association in the review or to conduct meta-analyses or other approaches to estimate summarized quantitative effects of these diets, limiting the strength of the findings. A rigorous systematic review would be needed to more clearly quantify and qualify the health effects from such diets. No librarian or information science professional was involved in the search strategy, thus limiting the breadth of this perspective. Given the length of time between the original literature search and publication, it is possible that more recent articles may have been published that were not captured in this perspective. Another limitation of this study is that it explored traditional diets primarily through a nutrition lens. In the broader interdisciplinary context of this topic, seminal texts from anthropology, archaeology, and culinary history may be missing from this perspective.

## Conclusions

Traditional diets across Asia, Latin America, Africa, and the African diaspora have in common an emphasis on whole grains, dark green and orange vegetables, and legumes, which have well-documented benefits for health. Many of these foods that are common in these traditional dietary patterns are less often consumed in Western diets. Thus, these healthy eating traditions deserve recognition and support wherever possible, including by public policies. When working with people of diverse racial and ethnic backgrounds, using culturally relevant models of healthy eating may be a successful strategy to promote personal and community wellbeing. The value of policies and programs that recognize and support traditional diets will benefit from more research on impacts of culturally tailored nutrition intervention programs.

## Author contributions

The authors’ responsibilities were as follows – KEL, SB-S, WCW: conceived the manuscript; KEL: wrote the first draft with contributions from WCW; KEL, SB-S, WCW, KJL, HC, KHKL, KLT, LHK: all edited, reviewed, commented on, and approved subsequent drafts of the manuscript; and all authors: read and approved the final manuscript.

## Conflict of interest

SB-S and KEL are employees of Oldways, a nonprofit nutrition education organization focused on cultural food traditions. Oldways created the Mediterranean Diet Pyramid, the Asian Heritage Diet Pyramid, the Latin American Heritage Diet Pyramid, and the African Heritage Diet Pyramid. KJL, HC, KHKL, KLT, LHK, and WCW serve as scientific advisors for Oldways.

## Funding

The authors reported no funding received for this study.
